# Family group conferencing for children and families: Evaluation of implementation, context and effectiveness (Family VOICE). Study protocol

**DOI:** 10.1371/journal.pone.0300834

**Published:** 2024-06-06

**Authors:** Jonathan Scourfield, Rhiannon Evans, Philip Pallmann, Stavros Petrou, Michael Robling, Kar-Man Au, Delyth Jones-Williams, Fiona Lugg-Widger, Melissa Meindl, Elizabeth-Ann Schroeder, Sophie Wood, David Wilkins

**Affiliations:** 1 Children’s Social Care Research and Development Centre (CASCADE), School of Social Sciences, Cardiff University, Cardiff, United Kingdom; 2 Centre for Development, Evaluation, Complexity and Implementation in Public Health Improvement (DECIPHer), United Kingdom; 3 Centre for Trials Research, Cardiff University, Cardiff, United Kingdom; 4 Nuffield Department of Primary Care Health Sciences, University of Oxford, Oxford, United Kingdom; Public Library of Science, UNITED KINGDOM

## Abstract

**Background:**

Family group conferences (FGCs) in child welfare bring immediate and wider family members together to decide on the best way to meet a child’s needs. Unlike professionally led meetings, the aim is for decisions to be made by or with family members. Qualitative and mixed-method research with FGC participants tends to show positive experiences: most participants feel their voices are heard; FGCs facilitate family-driven solutions and closer relationships—within families and with social workers. Although there is existing literature on FGCs, there is a paucity of robust comparative UK evaluations, i.e., randomised controlled trials or quasi-experimental studies. Comparative studies internationally have focused on a narrow range of outcomes, not recognised the importance of context, and paid little attention to the quality of delivery. Some qualitative studies have considered process and context but there is scant measurement of these. The aims of this study are, firstly, to establish how FGCs improve outcomes for families and what factors vary their quality, and, secondly, to assess longer-term outcomes in terms of service use and associated costs.

**Methods:**

Given the importance of process and context, evaluation informed by realist and complex systems approaches is needed. This multi-method evaluation includes a survey of FGC services in all UK local authorities (n = 212) to map service provision; co-design of programme theory and evaluation measures with family members who have experienced an FGC (n = 16–24) and practitioners (n = 16–24) in two sites; a prospective single-arm study of FGC variability and outcomes after six months; and comparison of service use and costs in FGC participants (n≥300 families) and a control group (n≥1000) after two years using a quasi-experiment.

**Discussion:**

This is a pragmatic evaluation of an existing intervention, to identify what mechanisms and contexts influence effective process and longer-term outcomes. The study is registered with Research Registry (ref. 7432).

## Introduction

A family group conference (FGC) in child welfare brings immediate and wider family members, and others from their social network, together to decide on the best way to meet the needs of a child who requires support and/or protection. Unlike professionally led meetings, FGCs aim to shift decision-making to family members. They do so more fully than attempts to make traditional case conferences more strengths-based [1]. Staff who are termed ‘co-ordinators’ prepare potential attendees for the FGC meeting and usually facilitate the start of the meeting, but then professionals leave the room so that family network members only can have ‘private family time’ [2]. Children and young people sometimes attend themselves, but this depends on age and the family situation [3]. Sometimes children and young people are represented by advocates. FGCs originated in Aotearoa New Zealand, with a focus on Māori whanau (families) and in the context of concern about the over-representation of Māori tamariki (children) in state child welfare interventions [4].

There is also concern in many other countries about rising levels of state intervention, especially rising rates of children in out-of-home care [5]. There is concern about a professionally led child protection system which can be confrontational in style [6] and focused on forensic investigation at the expense of support for families [7]. In this context, FGCs are often seen as an important element of developing a child welfare system that is based on more positive relationships between the state and vulnerable families [8].

FGCs have the two primary aims of increasing family participation in important decisions about children and reducing more intrusive state intervention.

FGCs are widely used in the UK. There are likely to be similarities and differences in policy and provision between the four nations (England, Scotland, Wales, Northern Ireland), but there is no recent comparative study that maps these differences. The Family Rights Group, who promote and quality assure FGCs across the UK, estimated in 2019 that three-quarters of local authorities in England and Wales run or commission an FGC service or are planning to do so [9]. We roughly estimate, based on the referrals expected for the randomised controlled trial run in England by Coram (a children’s charity with an evaluation arm), that around 15,000 children in the UK or more may be involved in FGCs each year [10]. This is a rough estimate only, based on local authority projections of how many pre-proceedings cases they expected during the Coram trial.

Qualitative and mixed-method research with FGC participants suggests that most families who have had an FGC feel that they offer an increased opportunity for their voices to be heard; hence, they can be seen to facilitate family-driven solutions [6, 11]. FGCs have also been found to help foster closer relationships between family members and improve partnership working between social workers and families [12]. Fewer studies have focused on the experiences of children specifically, but these have also reported positive experiences, as well as some challenges [2, 13].

It is important, however, to compare families who have attended FGCs with similar families who have not, to see how outcomes and experience differ. Several randomised controlled trials and quasi-experimental studies have been conducted internationally, with 18 included in a recent systematic review [14] of all types of participative family meetings (FGCs and other models with different names). Only two of the FGC studies included in this review took place in the UK [15], and all the FGC studies had a high or moderate risk of bias. Most focused on reduction in out-of-home care, showing mixed evidence of effectiveness. A similar systematic review for the Campbell Collaboration [16] also found mixed evidence for this outcome. Other outcomes are important, given that FGCs are designed to increase family involvement as an important goal in its own right [17]. Few comparative studies of any type of participative family meeting have considered outcomes such as family satisfaction, family empowerment or engagement with support services [7, 8] but the evidence on effectiveness for these outcomes is also mixed and of low quality. These comparative studies tend to focus on outcomes to the exclusion of consideration of process and context.

The contrast in findings between different types of studies is interesting. It might be explained by selection effects, e.g., if family members with a more positive experience are more likely to volunteer for qualitative interviews that can be time-consuming, compared to questionnaire completion, for example. It is quite likely explained by most qualitative studies being conducted only with families who have had FGCs, and not with similar families who have experienced conventional services but no FGC. Asking the same questions of families with similar problems who have not taken part in an FGC but had more conventional professionally led meetings makes for a more valid comparison of people’s experience of FGCs. Also, a critical perspective is needed on concepts and measures used in studies–for example, some measures used for family empowerment may be capturing parental self-efficacy more than ownership of key decisions about children.

To some extent, it is unsurprising that the outcomes of a complex intervention will be mixed. However, Nurmatov *et al*. [14] conclude that one possible explanation for mixed effectiveness is variation in the quality of delivery. They note that ‘it is likely that there is considerable variation in how well shared decision-making meetings, and indeed treatment as usual services, are actually delivered’ and they go on to say that ‘it is very likely that the passion and skill with which services are provided has a substantial impact on the difference’ [13, p.41]. They found that few studies in their review reported on fidelity to the shared decision-making model and none reported on the quality of delivery of usual services. They recommended that future studies include evaluation of the actual service delivery in addition to outcomes.

Some studies suggest that families who have had FGCs do not differentiate the process itself and their engagement with it from the outcomes they experience [18]. Mitchell [19] found that family members’ personal experience of the FGC process impacts their perception of the relevance of support offered, their sense of empowerment and their perspectives on outcomes achieved. The aspect of FGCs often thought to be the most important process issue, namely families’ rights to participation being upheld [18] can also be conceived of as an outcome [20]. Considering this, it is important to work with families who have lived experience of FGCs, to establish what, for them, are the important outcomes of FGCs, before assessing how well these outcomes are met in practice.

Despite there being a sizeable literature on FGCs to date, there remain several key limitations and evidence gaps. There is a paucity of robust comparative evaluations in the UK context. Comparative studies internationally have focused on a fairly narrow range of outcomes with minimal recognition of the contextual contingency of effects and little attention to the quality of service delivery. Some qualitative studies have considered process, but these have not involved measurement of context and process variables. The research evidence on FGCs demonstrates some of the complexity of delivering the intervention. In light of this, a more nuanced, complex systems approach to evaluation is needed.

Our study involves co-design with service users; has a comparative element; and measures context and process as well as outcomes in the short, medium, and longer term. The study aims to establish how FGCs improve outcomes for families and what factors vary their quality. To do so, the study poses the following research questions (RQs). The respective work packages (WPs) are noted, with these described in detail in the following sections.

- RQ1: What is the extent and nature of FGC provision for child welfare in the UK? (WP1)
- RQ2: How do families and practitioners see FGCs as impacting on families and what do they think might support or hinder their effectiveness? (WP1)
- RQ3: How does variation in the quality and implementation of FGCs impact on outcomes and acceptability? (WP2)
- RQ4: What are the longer-term outcomes of FGCs in terms of service use, e.g., child protection involvement and health care? How do these compare with similar families who have not taken part in FGCs and what are the economic consequences of FGCs? (WP3)

## Materials and methods

### Design and theoretical/conceptual framework

The study is informed by realist and complex systems thinking, integrating these into the well-established Medical Research Council approach for evaluating complex interventions, as demonstrated by Fletcher *et al*. [21]. Theorising intervention process and effects will move beyond the traditional, linear approach to modelling interventions and instead work with complex systems modelling, privileging context more clearly [22, 23]. In conceptualising context, we will draw on the framework of Pfadenhauer *et al*. [24].

It is a multi-method evaluation, including a survey to map service provision; qualitative co-design of programme theory and evaluation measures; a survey of family members at three time-points to assess process and self-reported outcomes; and a comparative study of longer-term service-use outcomes using linked routine administrative data. The study is registered with Research Registry (ref. 7432).

Project work packages:

- WP1: Identification and modelling of FGCs in the UK; modelling programme theory and development of evaluation design (RQ1, RQ2)
- WP2: Prospective single-arm study of FGC variability and outcomes after six months (RQ3)
- WP3: Comparison of service use and associated costs in FGC participants and a comparison group after two years using a quasi-experiment (RQ4)

### Work Package 1 –Identification and description of FGCs in the UK; modelling programme theory; and development of evaluation design (RQ1 and 2)

WP1 will focus on understanding the local variations of FGCs within the UK context and on modelling the programme theory of the FGC as an intervention–i.e., determining its key mechanisms and outcomes, and the implementation contexts that have an impact on its effectiveness. This will support the identification of the most appropriate evaluation measures.

Three key activities will be undertaken as part of WP1: identification and description of FGC services (A); modelling of FGC programme theory (B); and co-design and piloting of evaluation tools (C).

#### A. Identification of FGC services in the UK

We will conduct a survey of local authorities and their equivalents across the UK to identify the current use of FGCs, and differences in practice and contextual factors between authorities. The aim of the survey is to describe the FGC landscape in the UK. It will not provide enough depth to inform the programme theory (B below), but the information gathered about FGC practice and contextual differences will be used in combination with the programme theory to inform the development of the evaluation measures (C below). Results from the survey will also inform local authority selection for WP3.

*Sampling*. The sample frame will comprise all local authorities in England, Scotland and Wales, and Health and Social Care Trusts in Northern Ireland (n = 212). Given that most authorities are likely to have implemented a related service at some point, all will be included in the sample frame. It will also be helpful to know about local authorities who have either never used FGCs and those who have disinvested in them.

*Setting/context*. Local authorities have statutory responsibility for child welfare in England, Wales, and Scotland. In Northern Ireland the equivalent bodies are Health and Social Care Trusts, but for brevity, we use the term ‘local authorities’ here in reference to all UK nations. They both provide their own services and commission services from the private and third sectors.

*Data collection*. An online survey will be developed in collaboration with the Family Rights Group and in consultation with experienced FGC practitioners. There are two routes to reaching FGC services. Firstly, we will send the online survey link to all heads of children’s services in the UK and ask them to cascade the survey to relevant staff, such as FGC service managers. Secondly, local independent FGC services will also be identified directly via a systematic web search (‘family group conference’ and each local authority name). Identified FGC services will also be emailed the survey link and asked to take part. Consent will be sought via a tick-box on the first page of the questionnaire. The anticipated respondents are FGC service managers but there will be no criteria imposed as to who can complete the questionnaire.

The online questionnaire will be fairly brief and focused, including questions about:

- Whether or not FGCs are currently used in the local authority
- Whether FGCs were offered in 2017–19 (the baseline period for WP3)
- The stage of child welfare concern when FGCs are offered
- The family circumstances for which FGCs are used
- Which kind of organisation delivers the FGCs
- Whether there is any theoretical model that informs the approach used
- How services have been functioning in light of Covid-19
- Number of conferences run each year
- What data are currently recorded for evaluation purposes and how valuable this is
- Staff involved including contact details (for WP2)

There will be a mixture of fixed-response quantifiable questions and open questions with free text boxes.

Comparable surveys have achieved response rates ranging from 39% to 86% [25, 26]. Even a low response rate would mean a viable study, however, the main aim is to generate an understanding of different models and different types of implementation, rather than to achieve a highly representative sample. More important is diversity in context–data from different UK nations and different size local authorities–to capture variation. Additional aims are to make initial contact with practitioners who can form a community of practice for help in delivering WP2 and to identify local authorities where FGCs are not offered for WP3.

*Data analysis*. Descriptive statistics on survey responses will be produced using Stata software. Free text responses will be thematically synthesised.

#### B. Modelling of FGC programme theory

This element of the study will involve working with key stakeholders–families and practitioners–to co-design the FGC programme theory and appropriate outcomes. This will help inform the evaluation design.

Programme theory is the main output of a realist study and is defined as a ‘causal model linking programme inputs and activities to a chain of intended or observed outcomes, and then using this model to guide the evaluation’ [27, p.30]. Mechanisms, contexts, and outcomes are key concepts in realism and will be essential to identify to produce, test and refine a programme theory/theories. Mechanisms are underlying and often unobservable causal forces (e.g., how someone thinks and feels) that explain the observable outcomes [28] of FGCs. They are comprised of two parts–resources offered by a programme (like private family time in FGCs) to people, and how people respond or react to those resources [29]. Context is any pre-existing factor outside of the formal FGC intervention, that enables or inhibits the activation of programme mechanisms [27]. Context refers to any level of influence in a complex system, family problems, the FGC service, the local authority, or national policy that affects FGC provision.

We will conduct a series of consultations with stakeholders to generate an understanding of the overarching programme theory of how FGCs work in the UK, to construct a complex systems logic model and determine appropriate evaluation measures. This piece of work is framed primarily as stakeholder consultation on research design rather than data collection. The consultations will be preceded by a literature review of the international qualitative evidence on FGCs, with an emphasis on UK studies. Key databases will be searched including Medline, PsycInfo, Embase, Scopus, and CINAHL. These will be supplemented with grey literature searches. The identified literature will be explored for evidence of contexts, mechanisms and outcomes, and causal relationships between these, that will be brought together to produce a candidate programme theory and logic model, that will be presented to families and practitioners, and revised following their input.

*Sampling and research participants*. We have identified two FGC sites with contrasting contexts in which to conduct consultation workshops in London and North Wales. Both areas have FGC services with strong track records of service delivery and ongoing family involvement. These are the Camden Family Group Conference Service and Y Bont, based near Caernarfon. The two areas are contrasting in several respects. Camden is an inner city, multi-ethnic London borough. North Wales–Y Bont serves Gwynedd, Conwy, and Denbighshire local authorities–is a mixture of smaller towns and rural areas, mostly White and containing a high proportion of Welsh language speakers, especially in Gwynedd. Camden and North Wales both have pockets of extreme deprivation. From each of these areas we have recruited a peer researcher with experience of having taken part in FGCs as a family member. These staff were recruited with the help of FGC services in the two sites. They are full members of the research team and will take part in all aspects of WP1, but with particular responsibility for setting up and running the consultation with family members. They are supported by the CASCADE research centre’s framework for employing people with lived experience of social care.

Within each of these FGC services we will purposively sample a group of families who have received an FGC (n = 8–12 per site); and a group of practitioners, including local FGC co-ordinators, local social workers, and other FGC co-ordinators from across the UK (n = 16–24). The individuals from the two FGC sites will be identified and recruited with the help of the local FGC service and the peer researchers. Any family members, and any number of individuals from any one FGC will be eligible to take part, with no restriction in terms of recency of the FGC. However, families in the middle of legal proceedings about a child, will not be able to take part due to ethical reasons. The sample should ideally include a mixture of participants in terms of satisfaction with the FGC process. If more family members come forward than we can accommodate, we will explore their satisfaction with their FGC via response to a single question and purposively sample to achieve a range of satisfaction levels. There will be no restriction for professionals participating in terms of their level of experience or seniority. Emails and text messages will be sent by the two FGC services to families and coordinators who have taken part in FGCs, with information about the workshops. In recruiting participants, careful attention will be given to equality and diversity. Especially relevant diversity criteria are likely to be ethnicity and language use. We will ensure the consultation event is pitched appropriately for people with few educational qualifications and for ethnic minority participants who speak English as a second language. The workshops in North Wales will include use of both Welsh and English. The other FGC co-ordinators from across the UK will be identified through the survey of FGC services (see A. Identification of FGC services in the UK). Respondents will be asked if they would like to take part in other activities for this research project, and those who indicate interest will be emailed and invited to participate in the consultation workshops.

*Data collection*. In each site, we will host two in-person workshops for adult family members. We will also hold two online (video conference) workshops for children and young people across the two sites. Both adult and young person workshops will be three months apart, allowing for the research team to act on participants’ views following the first workshops and for sense-checking of actions the researchers have taken. There will also be two online workshops for practitioners, also three months apart.

Online workshops will be used to allow practitioners to attend from each of the UK nations, in addition to the two local sites. They will be used with young people for two reasons. One is the pragmatic consideration that young people may be more difficult to recruit, given that not all FGCs have children or young people attending, therefore merged workshops with young people from both sites together may be more realistic. The other consideration is that feedback from the pandemic service delivery experience was that many young people were positive about online communication, and this could make the meetings more likely to happen than in-person meetings [30].

The first round of consultations will focus on testing and refining the candidate programme theory/logic model, and the second on specific evaluation measures. The family events will be jointly run by the peer researchers and other members of the research team. In advance of the first consultation, the research team, including the peer researchers, will draw together data from the existing research evidence (reviewed qualitative studies and a recent realist review [31]) to develop initial candidate logic model(s) that depict the overarching programme theory for FGCs in the UK.

The presentation of the programme theory and logic model(s) will be discussed and agreed upon between the peer researchers and other members of the research team in advance of the first consultation events, with accessibility of language and concepts in mind. Through the consultations we will explore: 1) Does the candidate programme theory fit with the actual experience of being in an FGC? 2) How do FGCs impact on how people think and feel (mechanisms)? 3) What other things (contexts) affect how people experience FGCs (including the effect of Covid-19) and how successful they are? 4) What do good and poorly delivered FGCs look like? 5) What kinds of things do families have happen, or want to happen because of an FGC, that we should be measuring in an evaluation (outcomes)? For young people participating, recognising that this type of consultation may be less familiar to them, the agenda of the online workshops will include some lighter content as well as core FGC discussion points.

If recruited individuals do not want to participate in the group consultation, we will offer the opportunity to participate in an interview. Data will be recorded (audio for in-person meetings and video for online meetings) and transcribed verbatim. Written consent will be sought from participants 16 and over. For any children under 16, written consent will be sought by adults or carers with parental responsibility.

*Data analysis*. Ideas and notes generated through the consultations will be analysed for further evidence of contexts, mechanisms and outcomes (and causal relationships between these concepts), which will be used to confirm, refute or refine the initial candidate programme theory and logic model(s) to produce an overarching understanding of FGC practice in the UK, while recognising that not all data will be complementary, and a single unified understanding may be unlikely. Such discrepancies will be noted and explored more fully in the later stages of evaluation (e.g., semi-structured interviews in WP2). Consultation data will be analysed thematically [32] using a combination of a priori (e.g., theory, context, implementation, outcomes) and in vivo codes. The logic models will be revised so that they are more comprehensive and encompass context and implementation issues. These models will be accompanied by a narrative overview of the programme, which will provide a more detailed account.

There is a pragmatic compromise between maximum complexity and what can realistically be conveyed in a single-page summary model (and realistically measured in questionnaires–see below). It should be acknowledged that there is a risk that outcome indicators might become too homogenised, given the complexity of context and mechanisms. This is an inevitable limitation of a logic model that is concise enough to be useful to busy practitioners.

Acknowledging that the two case study sites could be considered enthusiasts for FGCs, we will use the WP1 survey of FGC services, which will cover a wider range of FGC practice across the UK, to contextualise our theorising of FGC process in context.

#### C. Identification and co-design of evaluation measures

We will build on elements A and B above to identify evaluation measures to operationalise our findings for the later stages of the evaluation. Evaluation tools (questionnaires) will be developed and piloted in WP1 to reflect the programme theory, including implementation, contexts and outcomes. Following a literature review of existing relevant measures and further consultation with families and practitioners, suitable scale measures will be selected for use in WP2. This will include a questionnaire for FGC participants at three time points–with existing measures adapted if necessary–and a questionnaire for FGC co-ordinators (i.e., staff who arrange and facilitate the conferences). The initial selection of potential measures will include peer researchers and options will be discussed at the second round of consultation events with family members, young people and practitioners. Some outcomes from the programme theory will be best measured longer-term using administrative data as part of WP3.

The problem of how to deal with multiple perspectives will be explored and resolved with family members and practitioners. Options include separately self-reported measures and a consensus score for the family unit.

The WP1 survey will identify FGC services across the UK and those opting for further involvement with the study will be invited to form a community of practice, to implement the use of the standardised evaluation questionnaires in routine practice in WP2. The questionnaire for FGC participants will be refined in consultation with this community of practice. The questionnaire will then be piloted with WP1 workshop participants and other family members recruited from the two WP1 local sites, using cognitive interviews to assess validity and likely feasibility. Cognitive interviews are a method for testing respondents’ understanding of survey response items. Participants are presented with the draft questionnaire and probes are used to check what they understand from the questionnaire wording. These cognitive interviews will include adult family members, young people, and some younger children (in connection with the accessible version of the families’ questionnaire). There will be approximately 20 interviews in total across all these groups.

The questionnaire for FGC co-ordinators will characterise the family situation, decisions made and the co-ordinator’s own rating of how effective the FGC was. The content of the co-ordinator questionnaire will link to the programme theory and questionnaire wording will be discussed in the second round of practitioner consultation workshops. It will also capture resource use and will be used as a basis for estimating the cost of delivering FGCs. The design of the resource use aspect will be informed by our previous systematic review of economic analyses of FGC 11 and it will be piloted to ascertain its acceptability, comprehension, and reliability.

A Families’ Research Advisory Group will also be set up, to meet biannually for the whole duration of the project (four years). This will be made up of adult family members with lived experience of participating in FGCs or of other aspects of involvement with children’s social care services. The acceptability of routine data follow-up for WP3 will be explored with members of this group, as well as the question of which outcomes discernible from these data sets should be assessed.

### Work Package 2: Prospective single-arm study of FGC variability and outcomes after six months (RQ3)

Public-facing title: Family views and context study (Family VOICE project)

WP2 considers process and short- and medium-term outcomes of FGCs, using evaluation tools (questionnaires) co-designed with families and practitioners in WP1. A questionnaire will be used with FGC participants, before, a few weeks after, and six months after an FGC.

#### Outcomes to be measured

Outcomes will be determined in collaboration with stakeholders in WP1. Candidate outcomes to be measured in this exploratory work package include the empowerment of FGC attendees two weeks and six months after the conference. In addition, the following outcomes will be explored: family communication, adult family member well-being, and perceived child safety and well-being (also reported by the child/young person if they have attended the FGC meeting and agree to take part in the study). None of these is of primary importance, but for the purposes of sample size calculation (i.e. the number of families needed to complete WP2 questionnaires), parental empowerment has been assumed to be the primary outcome.

#### Sampling, settings and research participants

WP2 involves working with a community of practice of FGC practitioners across the UK, identified in the WP1 survey of FGC services, to adopt the families’ questionnaire designed in WP1 into routine use. The research team will keep in regular contact with this community. Any child welfare FGC service that wants to take part in WP2 will be able to do so; there will be no exclusion criteria. Within and between services that volunteer to take part there should be variation in FGC use that will allow the impact of context to be considered. Within each service, any child welfare FGC would be eligible for inclusion. The only exclusion criterion would be FGCs convened because of adult social care needs, which have no child welfare dimension.

Participating FGC services will comprise a prospective single-arm study of the impact of complex systems on family outcomes six months after an FGC, using family self-report via a questionnaire and semi-structured interviews with a sub-sample. All family members who have participated in a conference will be invited to take part, with no lower age limit imposed. Those who give written consent to take part will have contact details recorded at the point of recruitment (baseline) and these will be used for subsequent follow-ups. Participants are likely to include extended family members as well as biological and social parents and children/young people. The co-ordinator will be asked to complete a questionnaire, but no other professional who has attended an FGC, such as a social worker or advocate.

As noted above, the evaluation tools will be co-designed with families during WP1, and outcomes and sample sizes will be decided during this co-design phase. To obtain an estimate of the scale of the study we assume a primary outcome of empowerment and a single composite score across all FGC participants, whilst acknowledging this may not prove to be a preferred measure. We further hypothesise there could be a difference in empowerment according to a characteristic of the FGC–e.g., higher or lower levels of participation or an FGC taking place at a late stage of concern or earlier.

Having consulted results from comparative studies of family group decision-making that have measured empowerment at later time points [33–35], it is possible to estimate a sample size for comparing different kinds of FGC (e.g., early help vs. edge of care–contexts where the level of empowerment could be quite different). To detect a 0.25 difference between two groups of equal size on a five-point scale, with a standard deviation of 0.50, 95% confidence and 80% power, a sample size of 128 families will be required (two-sample t-test). For a pre-post comparison to estimate the mean improvement from baseline to six months in all FGC participants, N = 128 will allow us to detect a minimum effect size of 0.125 with 80% power, or a minimum of 0.144 with 90% power (one-sample t-test). Assuming a 36% attrition rate between the first questionnaire and six-month follow-up, which is conservative in light of the same rate having been found by Dijkstra et al. over a 12-month follow-up period [34], an initial sample size of 200 FGCs will be needed. The current English trial of FGCs at pre-care-proceedings stage 6 shows an average of 73 conferences a year planned in each of the 24 participating local authorities, so for a minimum of 128 families to participate in the full survey across several different services seems feasible.

#### Data collection

We envisage survey data will be collected from families at three time points, although this timing will be discussed and agreed with families and practitioners in WP1 workshops:

- At baseline–i.e., before the FGC takes place–data collection will be challenging. Who will attend is often unpredictable in advance. Once participants have arrived for the FGC but before it starts, there are often difficult family dynamics, meaning that completion of a full questionnaire would probably not be feasible. We will assess in WP1 what is possible. Several administration methods will be considered, such as paper, phone, or online questionnaire, distributed via email, WhatsApp or text messaging.
- Follow-up a few weeks after the FGC will capture short-term impacts, in particular the initial family appraisal of the FGC experience and of decisions made. Mechanisms and context will be important to assess at this point from questionnaire responses.
- Medium-term impacts will be measured at six months, to capture enduring consequences of the FGC process for families. Some measures will be repeated from baseline and short-term follow-up data collection.

Questionnaire wording, determined through co-design in WP1, will be as simple as possible, bearing in mind a range of reading abilities. There will also be a shorter, accessible version for use with young children (e.g., under 11 years) and people with learning difficulties or low levels of literacy, which will also be co-designed with families and piloted, including checking with a learning disability charity. Administration methods will be determined during WP1, but at this point, we can envisage a choice for participants–e.g., online self-completion as the dominant method with researcher-supported data collection over the telephone where requested. We will provide specific training for researchers doing telephone questionnaires, with a script-based approach to maximise reliability.

Soon after each conference, the FGC co-ordinator will fill in a separate short questionnaire (also developed in WP1) to characterise the family situation, decisions made, rating of FGC effectiveness and resource use.

We will draw on the substantial evidence base for maximising response rates and retention [36, 37] and consult with families in WP1 about effective approaches. We will offer a financial incentive of £30 per respondent (£10 per questionnaire) to be paid shortly after completion of each questionnaire. Parkinson et al.’s review of incentives in clinical trials [38] found it was better to split payment between tasks and to make payment as soon as possible. Our recruitment and retention protocol will include a variety of methods to chase up non-response, including email and text message. We will ask participants for full contact details and permission to contact them via their preferred method when first recruited, and we will consider requesting secondary contact details (e.g. for a partner), if participants are happy to share these with us. A short practitioner-facing protocol will be developed to provide clear instructions to recruiting practitioners. Site-specific feedback will be an important incentive for services to support healthy recruitment. Follow-up at short- and medium-term time points will be done by the research team.

Semi-structured telephone interviews will be conducted with family participants, for further exploration of mechanisms and context. The interview schedule will not be drawn up until baseline questionnaire data are in, in order to pick up on emerging themes, but broadly the areas covered will be the same as in the questionnaire, though obviously in more depth and expressed in the participants’ own terms. We will use a purposive sample of five FGC participants from each of four services that participate in WP2 questionnaire data collection. We estimate this sample size to be sufficient to capture variation of context–e.g., stage of child welfare concern when FGCs take place and provision of services (i.e., by local authorities or by independent organisations). Participants willing to be interviewed will signal via the questionnaires and a purposive sample will be selected using variation of context as the main criterion. The interviews will be audio recorded and transcribed.

The intention is for the evaluation tools to become embedded into routine practice, enabling ongoing self-evaluation by services, an essential part of fostering a culture of service improvement. Ongoing support will be provided from research project staff to support the community of practice, with a view to it becoming self-sustaining after the project has finished.

Some important considerations for embedding the research tool into routine practice include co-designed content and data collection method (WP1); liaison with umbrella organisations such as the Family Rights Group, FGC networks for each of the four nations and the British Association of Social Workers FGC practitioners group; collecting no more data than families and services can tolerate; a real-time system for monitoring completion; ensuring feedback to people using services and FGC staff to show how data are being successfully collected; preparedness to be flexible, e.g., in terms of data collection mode preferences; and recruiting local champions for sites to support completion and contribute to problem solving.

We have explored the feasibility of routine use with FGC practitioners and one practical reality to consider is that funders often require some specific evaluation data. In the case of Y Bont, this is different in the three local authorities who commission their FGC service. Any questionnaires for families or practitioners developed in WP1 will need to be appropriately short and focused, as they may well have to be used alongside another type of data collection for local purposes. The logistics of this will be explored with services participating in WP2.

#### Data analysis

Descriptive summaries of outcomes, by time point, as well as simple pre-post comparisons (mean difference with two-sided 95% confidence interval) will be produced using Stata or R software. Contexts will be assessed per FGC, from a combination of family and practitioner questionnaire data. Additional analyses will involve fitting two-level regression models with random family effects to study the interplay of multiple contextual and implementation factors per follow-up time-point and longitudinally, as well as outcome variability between family members attending the same FGC. Fixed effects will include time-point, contextual/implementation factors, and possibly time-by-contextual factor interaction terms. Examples of possible contextual and implementation factors would be family size, stage of the child welfare process (e.g., early help, child in need, edge of care) and local authority culture, as assessed by family and practitioner (possibly a scale measure). Estimates of the effects of contextual factors will be presented with two-sided 95% confidence intervals and p-values.

Data from the WP2 family survey data a few weeks after FGC will be used to assess the reliability and validity of outcome measures by repeating or extending some of the original validation work on key measures, particularly where they have been adapted for use in this study, using e.g., Cronbach’s alpha, criterion validation and examination of factor structure and ceiling and floor effects. A statistical analysis plan will be developed to cover both WP2 and WP3.

Semi-structured interviews will be coded in NVivo software, using thematic analysis31. The coding frame will be developed through a combination of the programme theory from WP1 and inductive initial analysis of interview transcripts, with context, mechanisms and outcomes featuring as analytic codes.

### Work Package 3: Comparison of service use and associated costs in FGC participants and a comparison group after two years using a quasi-experiment (RQ4)

Public-facing title: Service use study (Family VOICE project)

WP3 is a quasi-experiment to assess service use outcomes of FGCs and the economic dimension of these. The experimental dimension is comparison of families who have had FGCs with similar families in local authorities with no FGC provision. This work package will assess some longer-term outcomes from the programme theory developed in WP1 for which administrative data are more suitable than self-report, because of the need for complete coverage of the relevant population without social desirability bias.

Outcomes to be measured include:

1. Primary outcome

The primary outcome is whether category (of seriousness) of children’s social care intervention at two years after FGC (or equivalent date for comparison group) is the same level, lower or higher than at the time of FGC. A ladder of escalation will be used, with state intervention categories ordered from most to least serious, including the child having been:

- categorised as in need (and reason for this);
- subject to a child protection investigation;
- on a child protection plan;
- looked after by the local authority (and, if so, on what legal basis; type of care–foster, kinship, residential, placed with parents).

The primary outcome variable will be binary, describing whether the category of statutory intervention at 2 years after FGC (or equivalent date for the non-FGC group) is the same or lower than at the time of FGC (or equivalent date) or whether it is higher.

2. Secondary outcomes

Highest category of children’s social care intervention since the FGC (or equivalent date for comparison group) at any time during the two-year follow-up.

A summary measure of duration and seriousness of all children’s social care intervention during follow-up.

Health care use for health conditions and events which might potentially be affected by FGC attendance–e.g., child and parent mental health, serious injury to a child, assault resulting from domestic abuse:

- hospital admissions;
- outpatient appointments;
- ‘Improving Access to Psychological Therapies’ [39] services;
- community mental health services;
- accident and emergency attendances.

Absence from school and special educational needs provision.

Incremental cost per adverse social-care-outcome-adjusted year avoided.

#### Sampling, settings and research participants

Families who have attended FGCs will be identified by local authorities–we will work with several large authorities in England, and access anonymised data from the Department for Education, NHS Digital and the Office for National Statistics (ONS). This part of the study is not linked in any way to the WP2 families. In these participating local authorities, we will also access routine data on families who have been referred to children’s services but have not had an FGC, in order to delineate the circumstances in which families do and do not take part in FGCs in these particular authorities.

A comparison group of local authorities which do not offer FGCs will be identified in the WP1 survey. Families from these authorities will be identified from the Children in Need Census. This part of the study uses data from only one UK country–England–because the datasets are different for each nation and given the complexity of data linkage it would not be feasible to attempt this study in more than one nation. We have chosen England as having the largest population.

Eligible families will be those with at least one child under 18 years of age referred to departments of Children’s Social Care Services from 1.1.17 to 31.12.19 at participating local authority sites in England (there is no intention to connect these local authorities to those taking part in earlier work packages). No additional exclusion criteria will be applied. At this stage we are not imposing a lower age limit. However, although we are confident that linkage to health records for school-age children is viable, there is some uncertainty about this linkage for pre-school children. We will therefore have to test the feasibility of extending the age profile to include all children from birth, in the early stages of WP3.

To make the two groups of families from different local authorities comparable for analysis, we will use statistical techniques to improve balance across the two groups for several known confounders. We will use propensity score weighting [40, 41] to estimate the average ‘causal’ effect of having an FGC on the outcomes.

A realistically achievable sample size of 300 families who have had an FGC and 1000 ‘control’ families in local authorities that do not offer FGCs would allow for the detection of a difference of 10.5 percentage points between groups for a binary outcome in a ‘worst-case scenario’ (i.e. proportions around 0.5) with 90% power and 95% confidence (two-sample test for proportions), but since propensity score weighting tends to shrink the ‘effective’ sample size the minimum detectable difference will be somewhat larger.

#### Data collection and linkage

Children referred to children’s services in applicable English local authorities (FGC providers and non-providers) during the intervention period (2017–19) will be identified from the Child in Need dataset within the National Pupil Database (NPD, Department for Education) which can be used to identify all school-aged children. This period has been selected on the basis of this being the most recent available where two-year follow-up in routine data is possible. The unique pupil ID can be used to link to other relevant NPD datasets, including Child Looked After, school attendance and special educational needs. Matching to Hospital Episode Statistics (HES) records will be undertaken using standard linkage strategies for HES and NPD data (e.g., using date of birth, gender, postcode) and quality of linkage assessed by estimating rates and distribution of linkage errors. Linkage will be achieved via the E-CHILD database [42]. Identifiers for FGC families will be supplied by local authorities. Health care records for birth mothers will also be matched in E-CHILD. The exact model for data linkage will be established in WP1. Anonymised, linked data will be accessed via the ONS Secure Research Service (ONS-SRS) and analysed by Cardiff and Oxford University staff via verified remote access.

We will follow up children in these datasets for two years following the date of referral. We have allowed for this work package to run throughout the four years of the project in recognition that permissions for data linkage and the delivery of linked data are very time consuming and often subject to delays. We also need to allow for follow-up. The latest follow-up data will be recorded in December 2021 and this data set will be first available to researchers in the Spring of 2023, two years into the project.

The Covid-19 lockdowns will influence the patterns of service provision from the end of March 2020, but this will be the same for families who have and have not taken part in an FGC between 2017 and 2019 and changes in service patterns will be interesting in their own right. Any difference between FGC and non-FGC families in post-lockdown trends can be noted. How Covid-19 and lockdowns affect the data will be considered when we write a definitive analysis plan.

#### Data analysis

We will use Stata or R software to estimate a family’s propensity for having an FGC with a logistic regression on variables including stage of the child welfare process (referral, child in need, child protection assessment, child protection plan), reason for referral and family demographics. The analysis model for the primary outcome variable (whether the level of social care intervention at two years after the FGC–or equivalent baseline date for the control group–has increased or not) will be two-level logistic regression, with random effects for local authorities and weighting of families according to their propensity for having an FGC. Fixed effects will include intervention (whether a family has had an FGC or not) as well as other factors–to be determined in WP1 –to account for the different ways FGCs are set up in different local authorities. Effect estimates will be presented with two-sided 95% confidence intervals and p-values. Secondary outcomes will be analysed using the same propensity score weighting approach and appropriate two-level regression models depending on the type of outcome variable (e.g., logistic for binary outcomes, linear for continuous outcomes).

We will undertake multiple imputation to explore the impact of potential missing data. We will also consider alternative covariate balancing or matching methods, such as entropy balancing [43], Mahalanobis distance matching [44] and coarsened exact matching [45] if propensity score weighting does not achieve good enough covariate balance and/or as sensitivity analyses. Detailed statistical analysis plans for WP2 and WP3 will be finalised prior to any analysis being performed, and these will be published on the study website.

#### Economic evaluation

The following health economic analyses will be conducted:

- Cost-consequences analysis with costs encompassing health care, social care and education, presented in a disaggregated manner.
- Cost-effectiveness expressed as incremental cost per additional case of child protection or out-of-home care avoided.

the economic evaluation of FGCs will be conducted from a public sector perspective. Primary micro-costing will be undertaken in WP2 via the practitioner questionnaire to estimate the cost of delivering FGCs across service model delivery types and geographical settings, including the costs of the identification and referral processes, training of accredited providers, delivery of sessions in alternative formats, participant monitoring activities and any follow-up/management. Where applicable the unit costs of resource inputs will be estimated in accordance with the principles of opportunity costs.

Broader health care, social care and education resource use over the follow-up period will be extracted from HES and NPD records and costed using national reference cost schedules. Resource use will be measured by the number and where possible, the duration of admissions, appointments and attendances during the 2-year follow-up period. Mean differences in children’s health and social care use and special educational needs provision and their associated costs (between the intervention and control arms) will be estimated using t-tests and bootstrap 95% confidence intervals that will be computed based on 1,000 (or more) replications.

The primary objective of the health economic evaluation is to estimate the incremental cost per additional case of child protection or out-of-home care avoided. Bivariate regression of costs and consequences, with multiple imputation of missing data (e.g., as a result of loss to follow-up), will be conducted to generate within-study estimates of incremental cost-effectiveness associated with FGCs.

A cost-consequences analysis will additionally be conducted, and we will present disaggregated costs and disaggregated consequences (outcomes 1 to 3 above), together with the estimates of the mean costs of the comparator interventions with appropriate measures of dispersion. Cost-consequences analyses are recommended for complex interventions that may have multiple implications [46], and for public health interventions which may have an array of benefits that are difficult to synthesise in a common unit [47].

Our analytical strategy will be informed by recent guidance on accounting for selection biases within economic evaluations using individual patient level observational data [48]. A key methodological challenge will involve generating expressions of cost-effectiveness amenable to broader cost-effectiveness comparisons by decision makers. A range of economic values for avoiding adverse child welfare outcomes will be informed by a review of the revealed and stated preference literature related to children’s social care, as well as by WP1. A series of sensitivity analyses will be undertaken to explore the implications of uncertainty on the incremental cost-effectiveness ratios.

**Fig 1:**
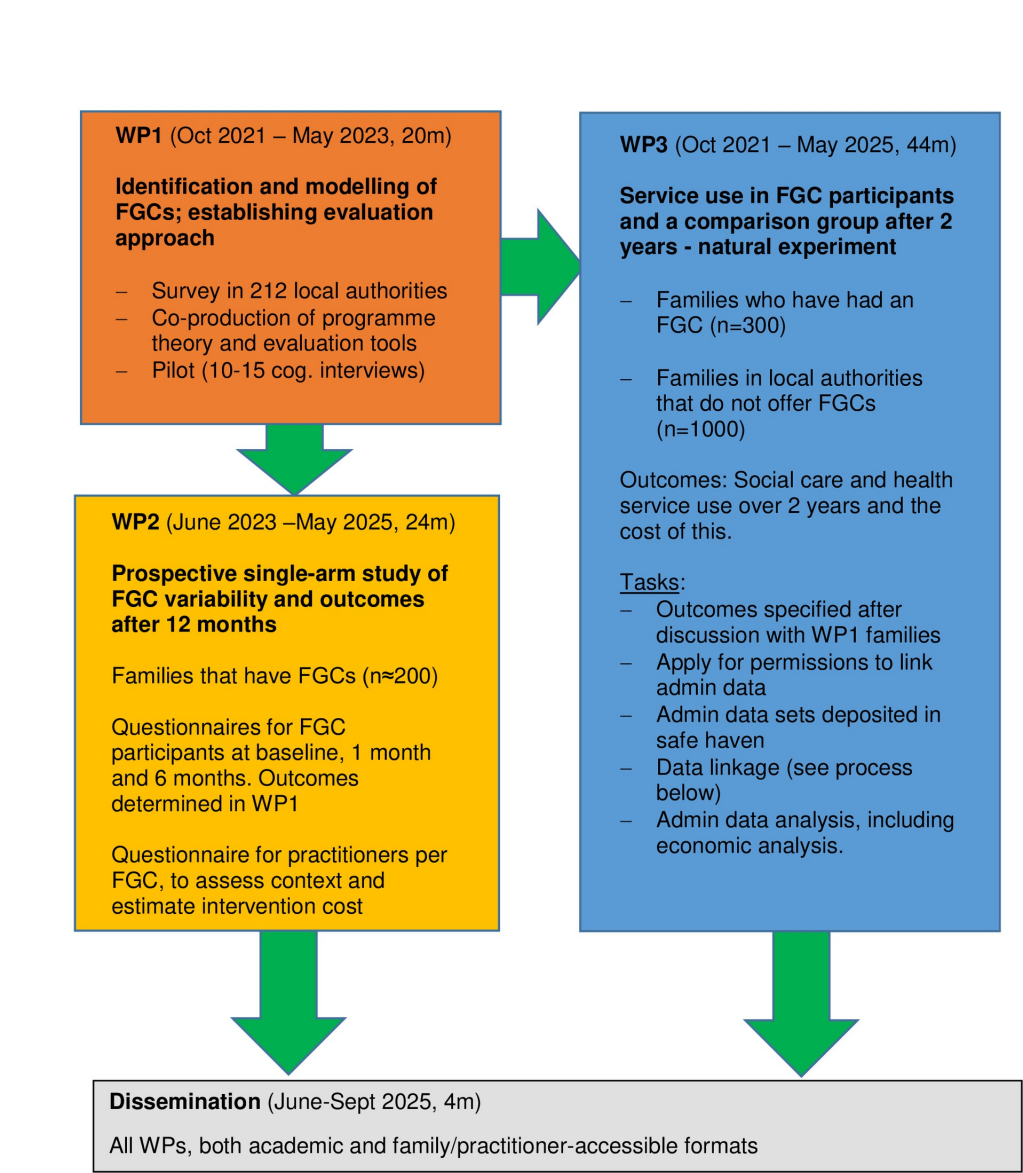
Flow chart of study work packages.

Fig 1 illustrates the relationship between the three work packages. They connect insofar as WP2 and WP3 each assess different elements of the programme theory developed in WP1. Family-reported mechanisms, context and outcomes will be assessed in WP2. Outcomes related to service use (e.g., reduced state intervention to prevent child maltreatment) and some aspects of context (e.g., local authority) will be assessed in WP3.

### Ethics and governance

All elements of WP1 have ethical approval from the UK Health Research Authority’s Social Care Ethics Committee (REC reference 22/IEC08/0003). WP2 has ethical approval from the UK Health Research Authority’s Social Care Ethics Committee (REC reference 3/IEC08/0029). WP3 has been approved by the South West–Cornwall and Plymouth Research Ethics Committee (REC reference 22/SW/0144). Approval has been given for the whole study by the Association of Directors of Children’s Services Research Group for England and initial support from the Northern Ireland Health and Social Care Board. No such national approval exists in Wales or Scotland.

Local authority records will be accessed without consent under General Data Protection Regulation (GDPR) Article 6(1). We consider this research to be of public interest. The proposed WP3 data flow from LAs to DfE is already covered by existing legal remits (under section 83 of the Children Act 1989. All individual-level linked project data will be pseudonymised and stored at the ONS-SRS.

A Project Steering Committee has been established to bring independent academic and sectoral expertise. The Chair is an experienced social work academic, and the committee includes methodologists, a statistician, a representative from the Family Rights Group who quality assure FGCs, and a civil servant working in national child and family policy. It will meet once a year throughout the project. As mentioned earlier, a Families’ Research Advisory Group will also run throughout the project, in parallel to the Project Steering Committee.

## Discussion

The study is an example of a pragmatic evaluation of an existing intervention, to identify what mechanisms and contexts influence effective process and longer-term outcomes [49]. Its strengths are the inclusion of an element of co-design with both family members and practitioners, and comparisons, both internally, within a pool of FGCs from different contexts, and externally, with families who did not have FGCs because they were not offered in their areas. Limitations include consulting with families in only two UK sites in WP1, the lack of a comparison group in WP2, and the potential for baseline differences in unknown or unmeasured confounders between the two groups being compared in WP3.

The study’s original contribution will be helping to explain variation in family-reported outcomes in terms of their relationship with mechanisms and context (WP2); and one of the longest follow-up periods of any FGC study (WP3), using administrative data with good quality coverage and fewer selection issues than in questionnaire studies. Findings from these work packages should be of use for policy and practice, as well as the programme theory developed in WP1, the mapping of UK services, and the WP2 questionnaires that will be available for future use in routine practice.

It is worth noting some unexpected practical barriers encountered, for other researchers undertaking UK-wide studies in this field. Firstly, it is important for social care researchers to note that the Northern Irish social care system is joint with the health service. This means that in applying for ethical approval, it is in fact incorrect to describe a UK-wide social care research project as not taking place in the NHS, even though it is not assessing a health care intervention, and even where no NHS data are involved (as for our WP1). In the case of Northern Ireland, the setting is classed as both social care and NHS because of the structure of services. There are additional processes and permissions associated with research in the NHS that some social care researchers may not be familiar with.

To approach individual social services departments in Wales and Scotland, there is no national prior approval needed. In England and Northern Ireland there are additional national processes, but these have different outputs. In England, approval from the Association of Directors of Children’s Services gives researchers permission to contact individual local authorities, although there is a cost attached–a sliding scale depending on the size of the research grant. In Northern Ireland, initial approval is needed from the Northern Irish Health and Social Care Board, but this does not in fact constitute approval to approach each local trust. Each one must agree this separately and the process is similar to the NHS research governance process in the rest of the UK.

Another barrier that is useful for other research teams to know about is that the UK National Social Care Research Ethics committee took the view that parents involved in care proceedings should either be excluded from WP1 workshops, or it should be explained to them that anything they said could be used in court. Considering this stance, we decided to exclude this group of parents, on the basis that the context of family court proceedings might make discussion about their circumstances emotionally difficult. However, the idea that any involvement of such parents in research would mean their words could be used in court has wider implications that need discussion with the committee and within the community of social care researchers.

It should be acknowledged that recruitment to the ‘Family views and context study’ (WP2) will be challenging, as it relies on multiple co-ordinators in several different FGC services recruiting multiple family members. As noted earlier, a recruitment and retention protocol, informed by evidence on successful strategies, will be used to support the process, including the use of financial incentives. It is nonetheless possible that in some services insufficient numbers of families will be recruited and small sample sizes will preclude site-specific feedback.

Any essential amendments to the protocol will be agreed by the Project Steering Group and research funders and then published on the funder’s study website.

## References

[1] Appleton J.V., Terlektsi E., and Coombes L. (2015). Implementing the Strengthening Families approach to child protection conferences, British Journal of Social Work, 45, 5, 1395–1414.

[2] Merkel-Holguin L. (2004). Sharing power with the people: family group conferencing as democratic experiment. Journal of Sociology and Social Welfare, 31(1), 155–174.

[3] Holland S. and O’Neill S. (2006) ‘We had to be there to make sure it was what we wanted’. Enabling children’s participation in family decision-making through the family group conference, Childhood, 13 (1): 91–111

[4] Ban P. (2005) Aboriginal child placement principle and family group conferences, Australian Social Work, 58:4, 384–394.

[5] Family Rights Group (2018) Care Crisis Review—Options for change. London, Nuffield Foundation.

[6] Forrester D., Kershaw S., Moss H. and Hughes L. (2008), Communication skills in child protection: how do social workers talk to parents? Child and Family Social Work, 13: 41–51.

[7] Featherstone B., White S. and Morris K. (2014) Reimagining Child Protection. Towards Humane Social Work with Families, Bristol, Policy Press.

[8] Mason P. with Ferguson H., Morris K., Munton T. and Sen R. (2017) Leeds Family Valued. Evaluation report. Department for Education.

[9] Family Rights Group (2019) Family Group Conferences and Lifelong Links, https://web.archive.org/web/20190101000000*/https://www.frg.org.uk/involving-families/family-group-conferences

[10] Coram (2020) Protocol for a randomised controlled trial of Family Group Conferencing at pre-proceedings stage. What Works for Children’s Social Care.

[11] Holland S., Scourfield J., O’Neill S., & Pithouse A. (2005). Democratising the family and the state? The case of family group conferences in child welfare. Journal of Social Policy, 34, 59–77.

[12] Pennell J and Burford G. (2000) Family group decision making: Protecting children and women. Child Welfare Vol LXXIX, 2: 131–158. 10732256

[13] Bell M. and Wilson K. (2006) Children’s views of family group conferences, The British Journal of Social Work, 36, 4: 671–681.

[14] Nurmatov UB., Foster C, Bezeczky Z, Owen J, El-Banna A, Mann M, et al. (2020) Impact of shared decision-making family meetings on children’s out-of-home care, family empowerment and satisfaction. A systematic review. What Works for Children’s Social Care.

[15] Munro E. R., Meetoo V., Quy K. and Simon A. (2017) Daybreak Family Group Conferencing: children on the edge of care. Department for Education.

[16] McGinn T., Best P, Wilson J, Chereni A., Kamndaya M. and Shlonsky A. (2020) Family group decision‐making for children at risk of abuse or neglect: A systematic review. Campbell Systematic Reviews; 16:e1088. doi: 10.1002/cl2.1088 37131917 PMC8356301

[17] Morris K. and Connolly M. (2012) Family decision making in child welfare: challenges in developing a knowledge base for practice. Child Abuse Review 21: 41–52.

[18] Frost N., & Stein M. (2009). Editorial: Outcomes of integrated working with children and young people. Children and Society, 23, 315–319.

[19] Mitchell M. (2020) Reimagining child welfare outcomes: Learning from Family Group Conferencing. Child and Family Social Work. 25:211–220.

[20] What Works for Children’s Social Care (2020) Outcomes framework https://whatworks-csc.org.uk/research/outcomes-framework-for-research/

[21] Fletcher A., Jamal F., Moore G., Evans R.E., Murphy S. and Bonell S. (2016) Realist complex intervention science: Applying realist principles across all phases of the Medical Research Council framework for developing and evaluating complex interventions. Evaluation 22(3): 286–303. doi: 10.1177/1356389016652743 27478401 PMC4946011

[22] Rehfuess EA, Booth A, Brereton L, Burns J, Gerhardus A, Mozygemba K, et al. (2018) Towards a taxonomy of logic models in systematic reviews and health technology assessments: A priori, staged, and iterative approaches. Research Synthesis Methods. 9(1):13–24. doi: 10.1002/jrsm.1254 28677339

[23] Mills T., Lawton R., Sheard L. (2019) Advancing complexity science in healthcare research: the logic of logic models. BMC Medical Research Methodology. 19(1):55. doi: 10.1186/s12874-019-0701-4 30871474 PMC6419426

[24] Pfadenhauer L.M., Gerhardus A., Mozygemba K. et al. Making sense of complexity in context and implementation: the Context and Implementation of Complex Interventions (CICI) framework. Implementation Science 12, 21. doi: 10.1186/s13012-017-0552-5 28202031 PMC5312531

[25] Corliss C., Addis S., El-Banna A., Maxwell N., Scourfield J., Warner N., et al. (2021) The views of local authorities in England on how to prevent children being in care. Child Care in Practice, awaiting issue.

[26] Baginsky M., Ixer G. and Manthorpe J. (2021) Practice frameworks in children’s services in England: An attempt to steer social work back on course? Practice, 33, 1: 3–19.

[27] Rogers P.J. (2008) Using programme theory to evaluate complicated and complex aspects of interventions. Evaluation, 14(1): 29–48

[28] Pawson R., & Tilley N. (1997). An introduction to scientific realist evaluation. In Chelimsky E & Shadish W. R (Eds.), Evaluation for the 21st century: A handbook (pp. 405–418). London, Sage.

[29] The RAMESES II Project (2017). What is a mechanism? The RAMESES II Project. www.ramesesproject.org

[30] Roberts L., Rees A., Mannay D., Bayfield H., Corliss C., Diaz C., et al. (2021). Corporate parenting in a pandemic: Considering the delivery and receipt of support to care leavers in Wales during Covid-19, Children and Youth Services Review, 128, 106155 doi: 10.1016/j.childyouth.2021.106155 36540703 PMC9756298

[31] Stabler L., O’Donnell C., Forrester D., Diaz C., Willis S, and Brand S. (2019). Shared decision-making: What is good practice in delivering meetings? Involving families meaningfully in decision-making to keep children safely at home: A rapid realist review. What Works for Children’s Social Care.

[32] Braun V. & Clarke V. (2006). Using thematic analysis in psychology. Qualitative Research in Psychology, 3, 77–101.

[33] Sheets J., Wittenstrom K., Fong R., James J., Tecci M., Baumann D.J., et al. (2009) Evidence-based practice in family group decision-making for Anglo, African American and Hispanic families, Children and Youth Services Review, 31, 11, 1187–1191.

[34] Dijkstra S., Creemers H.E., van Steensel F.J.A. et al. (2018) Cost-effectiveness of Family Group Conferencing in child welfare: a controlled study. BMC Public Health 18, 848. doi: 10.1186/s12889-018-5770-5 29986690 PMC6038335

[35] Dijkstra S., Asscher J.J., Dekovic M., Stams G.J.J.M. and Creemers H.E. (2018) A randomized controlled trial on the effectiveness of family group conferencing in child welfare: effectiveness, moderators, and level of FGC completion. Child Maltreatment, 24, 2: 137–151. doi: 10.1177/1077559518808221 30463425

[36] Edwards P., Roberts I., Clarke M., DiGuiseppi C., Pratap S., Wentz R., et la. (2002) Increasing response rates to postal questionnaires: systematic review. British Medical Journal 324, 7347, 1183. doi: 10.1136/bmj.324.7347.1183 12016181 PMC111107

[37] Fan W. and Yan Z. (2010) Factors affecting response rates of the web survey: A systematic review, Computers in Human Behavior, 26, 2, 132–139.

[38] Parkinson B., Meacock R., Sutton M. et al. (2019). Designing and using incentives to support recruitment and retention in clinical trials: a scoping review and a checklist for design. Trials 20, 624 (2019). doi: 10.1186/s13063-019-3710-z 31706324 PMC6842495

[39] NHS England (2022) Adult Improving Access to Psychological Therapies programme. https://www.england.nhs.uk/mental-health/adults/iapt/

[40] Li F., Morgan K.L. & Zaslavsky A.M. (2018) Balancing Covariates via Propensity Score Weighting, Journal of the American Statistical Association, 113:521, 390–400.

[41] Markoulidakis A. Taiyari K., Holmans P., Pallmann P., Busse M., M D. & Griffin B.A. (2022) A tutorial comparing different covariate balancing methods with an application evaluating the causal effects of substance use treatment programs for adolescents. Health Services Outcomes and Research Methodology. doi: 10.1007/s10742-022-00280-0 37207016 PMC10188586

[42] Mc Grath-Lone L., Libuy N., Harron K., Jay M.A., Wijlaars L., Etoori D., et al. (2022) Data Resource Profile: The Education and Child Health Insights from Linked Data (ECHILD) Database, International Journal of Epidemiology, 51 (1), 17–17f. doi: 10.1093/ije/dyab149 34788413 PMC8856003

[43] Hainmueller J (2012) Entropy balancing for causal effects: a multivariate reweighting method to produce balanced samples in observational studies. Political Analysis, 20(1), 25–46.

[44] Rubin DB (1980) Bias reduction using Mahalanobis-metric matching. Biometrics, 36(2), 293–298.

[45] Iacus SM, King G, Porro G (2012) Causal inference without balance checking: coarsened exact matching. Political Analysis, 20(1), 1–24.

[46] Drummond M. Sculpher M. Torrance G. O’Brien B., Stoddart G. (2005) Methods for the Economic Evaluation of Health Care Programmes. Oxford, Oxford University Press.

[47] National Institute for Health and Care Excellence (2013) Guide to the methods of technology appraisal (2013) Process and methods. www.nice.org.uk/process/pmg9 27905712

[48] Kreif N, Grieve R, Sadique MZ. (2013) Statistical methods for cost-effectiveness analyses that use observational data: A critical appraisal tool and review of current practice. Health Economics; 22(4):486–500. doi: 10.1002/hec.2806 22447531

[49] Evans R., Scourfield J., Murphy S. (2015). Pragmatic, formative process evaluations of complex interventions and why we need more of them. Journal of Epidemiology and Community Health, 69 (10), 925–926. doi: 10.1136/jech-2014-204806 25480407

